# Contamination of Aflatoxins Induces Severe Hepatotoxicity Through Multiple Mechanisms

**DOI:** 10.3389/fphar.2020.605823

**Published:** 2021-01-11

**Authors:** Zhenglai Hua, Rui Liu, Youwen Chen, Guangzhi Liu, Chenxi Li, Yurong Song, Zhiwen Cao, Wen Li, Weifeng Li, Cheng Lu, Yuanyan Liu

**Affiliations:** ^1^ School of Chinese Materia Medica, Beijing University of Chinese Medicine, Beijing, China; ^2^ Institute of Basic Research in Clinical Medicine, China Academy of Chinese Medical Sciences, Beijing, China

**Keywords:** aflatoxins, hepatotoxicity, mitochondria, miRNA, cell death, cell survival pathway, inflammation

## Abstract

Aflatoxins (AFs) are commonly contaminating mycotoxins in foods and medicinal materials. Since they were first discovered to cause “turkey X” disease in the United Kingdom in the early 1960s, the extreme toxicity of AFs in the human liver received serious attention. The liver is the major target organ where AFs are metabolized and converted into extremely toxic forms to engender hepatotoxicity. AFs influence mitochondrial respiratory function and destroy normal mitochondrial structure. AFs initiate damage to mitochondria and subsequent oxidative stress. AFs block cellular survival pathways, such as autophagy that eliminates impaired cellular structures and the antioxidant system that copes with oxidative stress, which may underlie their high toxicities. AFs induce cell death via intrinsic and extrinsic apoptosis pathways and influence the cell cycle and growth via microribonucleic acids (miRNAs). Furthermore, AFs induce the hepatic local inflammatory microenvironment to exacerbate hepatotoxicity via upregulation of NF-κB signaling pathway and inflammasome assembly in the presence of Kupffer cells (liver innate immunocytes). This review addresses the mechanisms of AFs-induced hepatotoxicity from various aspects and provides background knowledge to better understand AFs-related hepatoxic diseases.

## Introduction

AFs produced by *Aspergillus flavus* and *Aspergillus parasiticus* fungi are a class of polysubstituted difuranocoumarin compounds that exhibit mutagenic, teratogenic and immunosuppressive effects in farm and laboratory animals, and primarily invade the liver to produce serious hepatotoxicity (Mary et al., 2012; Wogan et al., 2012; Lin et al., 2014). AFs are commonly occurring mycotoxins that are classified into diverse types, such as AFB_1_, B_2_, G_1_, and G_2_. AFB_1_ is the most strongly hepatotoxic compound and hepatic carcinogen, and it is classified as a Group I carcinogenic agent by the International Agency for Research on Cancer (IARC) (Baan et al., 2009). Epidemiological investigations and experimental data indicate that dietary exposure to AFs is an important risk factor for hepatocellular carcinoma (HCC), and HBV infection combined with AFs exposure causes hepatotoxicity in areas of high exposure, such as South Asia, which increases the risk of HCC (Liu et al., 2012; Chen et al., 2019b). AFs contamination is prevalent in warm humid climates and irrigated hot deserts, which is serious for people’s health and economic aspects. Approximately 4.5 billion people around the worldwide are exposed to AFs-contaminated foods (Hamid et al., 2013). And the United States Food and Drug Administration (FDA) considers AFs as an inevitable contaminant of foods. So AFs are strictly banned in raw foods, processing products as well as herbal medicines worldwide. The European Pharmacopeia (EP) enacts strict limits for the presence of AFs in phytomedicines, including 2 μg/kg for AFB_1_ and 4 μg/kg for total AFs. Germany uses the same standard in any materials used in manufacturing of medicinal products (Zhang et al., 2018). Therefore, there is an absolute need to understand AFs contamination from various aspects, for example, how AFs contaminate foods and herbal medicines, how to rapidly and accurately detect AFs, and more importantly, how AFs threaten human health by inducing hepatotoxicity.

Fungi that produce AFs generally infect crops and spices, including wheat, corn, cotton and nuts (Kumar et al., 2016). Ruminants eating AFs-contaminated foods metabolize the toxins and excrete AFM_1_ in milk, which induces pollution in the transfer from crops into by-products and subsequent consumers (Prandini et al., 2009). Contamination of natural products and relevant by-products is divided into two phases: infection of developing crops in the first phase and any stage from maturation until consumption in the second phase (Cotty and Jaime-Garcia, 2007). Damaged susceptible crops and climate during the developing phase, crops caught by rain prior to or during harvest, and temperature and humidity during the storage phase are vital influencing factors for AFs contamination (Cotty and Jaime-Garcia, 2007; Kumar et al., 2016). Therefore, pre- and post-harvest management are critical practices for minimizing AFs contamination (Mahuku et al., 2019; Pandey et al., 2019). For nutrients in different grains, lipids from ground substrates significantly increase the risk of AFs production, and other nutrients, such as sugars and proteins, also are associated with AFs production (Liu et al., 2016). In contrast, microbial degradation of AFs has unique superiorities in the detoxification of AFs because of biodegradation mechanisms via modification of the structure of AFs or AFs absorption, which is an environmentally friendly and lower cost strategy (Verheecke et al., 2016; Afshar et al., 2020). Notably, ultraviolet irradiation is an effective method for reducing or detoxifying AFs via modification of a double bond in the terminal furan ring and breaking of the lactone ring, which may be toxicological sites in AFs (Mao et al., 2016).

Because AFs are derived from nature and structurally stable, contamination of herbal medicines caused by unfavorable environmental conditions may unavoidably occur in the field or at any stage in the supply chain, such as collection, processing, transportation, and storage. Different medicinal parts (seeds, roots, etc.) of medicinal materials that contain more oils, proteins, starches and sugars, such as *Platycladi Semen*, *Nelumbinis Semen* and *Polygalae Radix*, have higher risks of AFs contamination. Some animal medicines, such as *Mylabris and Hirudo*, that have a high content of proteins are also more susceptible to AFs contamination. AFs are also transferred into decoctions, resulting in a potential risk to consumers, especially whom take the herbal medicines directly or have a high consumption rate (Nian et al., 2018; Zhang et al., 2018; Raysyan et al., 2020). Notably, adverse reactions and toxicities of herbal medicines may originate from this type of pollution rather than the herbs themselves.

Because of the strong toxicity of AFs, it is not recommended to set a safe tolerance level or non-toxic dose internationally. The less we ingest, the safer we are. Therefore, methods to detect AFs rapidly, accurately and economically at a very low concentration are important for the safe use of foods and herbal medicines with complex substrates. Chromatographic methods, including thin-layer chromatography, high-performance liquid chromatography (HPLC) tandem mass spectrometry, fluorescence detector (FLD) and ultraviolet absorption detection (peak absorbance occurring at 360 nm) are used to detect AFs after extraction and clean up procedures (Ventura et al., 2004; D'Ovidio et al., 2006; Turner et al., 2009; Kong et al., 2013; Wen et al., 2013; Zhang et al., 2018). HPLC-FLD is widely used for AFs determination and is recommended in many counties because of its high sensitivity and selectivity (D'Ovidio et al., 2006; Kong et al., 2013; Wen et al., 2013; Zhang et al., 2018). Rapid screening techniques as qualitative assays, have important practical meanings in the determination and quality control of foods and medicinal materials for a large number of samples because of the simplicity of sample preparation, relatively inexpensive equipment and simple performance. Enzyme-linked immunosorbent assay, lateral flow immunoassay, gold immunochromatographic assay and aptamer-based lateral flow assay, which are mostly based on interactions between antibodies and antigens, are used to screen for the existence of AFs. However, techniques to overcome interference from the complicated matrix for the determination of trace AFs are needed (Hu et al., 2018; Zhang et al., 2018; Raysyan et al., 2020).

AFs inevitably exist in foods and herbal medicines and are one of the major causes of hepatic steatosis, necrosis, eventual cirrhosis and HCC (Jaeschke et al., 2012). The carcinogenic effects of AFs have been studied and summarized in the induction of liver cancer through forming deoxyribonucleic acid (DNA) adducts and modifying the tumor suppressor TP53 gene (Eaton and Gallagher, 1994). However, the toxic mechanisms of AFs and the impact of AFs on the structure and function of hepatocytes are not unclear. Therefore, an understanding about the mechanisms of AFs-induced hepatotoxicity and corresponding detoxification is meaningful. AFB_1_ has been extensively studied, which is a highly potent and toxic mycotoxin and the major product of AFs. We highlight the pathways of AFs-induced hepatotoxicity from the following aspects: metabolization to initiate toxicity, damage mechanisms in mitochondria, influence on the cell cycle and growth, induction of cell death, inhibition of cellular protective pathways and exacerbation of hepatotoxicity via inflammatory response.

## The Metabolic Bioactivation of AFs

**FIGURE 1:**
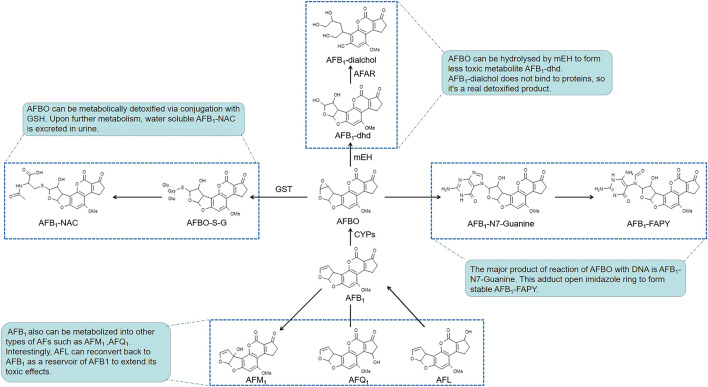
The bioactivation and related detoxified pathways of AFs.

After AFs are absorbed into hepatocytes, their toxicities are dependent on the balance of bioactivation and detoxification procedures (Figure 1). Cytochrome P450s (CYPs), which are a large superfamily of heme-binding enzymes, play an essential role in the synthesis and metabolism of endogenous substrates and in the biotransformation of xenobiotics. Isoenzymes of hepatic microsomal monooxygenases CYPs including CYP1A2 and CYP3A4 are needed to activate AFB_1_ to the extremely poisonous AFB_1_-8,9-epoxide (AFBO) in humans (Dohnal et al., 2014; Deng et al., 2018). Epoxide metabolites of AFs attack DNA via binding to guanine residues at the N7 position to form AFB_1_-N7-Gua adducts, which open the imidazole rings to form the more stable AFB_1_ formamidopyrimidine (AFB_1_-FAPY) adducts (Lin et al., 2014; Zhu et al., 2015). Other phase II enzymes, such as microsomal epoxide hydrolase (mEH) and aflatoxin-aldehyde reductase (AFAR), regulate the metabolism of AFB_1_. mEH converts AFBO into AFB_1_-8,9-dihydrodiol (AFB_1_-dhd), which is less toxic. However, AFB_1_-dhd still binds to lysine residues on proteins. Indeed, AFB_1_-lysine adducts in serum were significantly increased after exposure to AFB_1_ (Li et al., 2019). Subsequently, AFAR metabolizes AFB_1_-dhd to form AFB_1_-dialcohol, which is a real non-toxic product because AFB_1_-dialcohol dose not bind to proteins (Dohnal et al., 2014; Deng et al., 2018; Rushing and Selim, 2019). AFB_1_ is also converted to other intermediates, such as aflatoxicol (AFL), AFB_2a_, AFQ_1_ (a major product of CYP3A4 enzymatic action), and AFM_1_ produced by CYP1A2, etc. Interestingly, AFL is reconverted back to AFB_1_ as a reservoir of AFB_1_ to extend its toxic effects (Ayed-Boussema et al., 2012; Rushing and Selim, 2019).

Notably, AFs induce the expression of diverse CYPs. This induction may aggravate genetic and cellular toxicity by enhancing the process of bioactivation (Ayed-Boussema et al., 2012; Mary et al., 2015; Sun et al., 2015b; Gan et al., 2018; Muhammad et al., 2018a). Nuclear transcription receptors that regulate the expression of CYP1A, CYP2B and CYP3A subfamilies comprise the aryl hydrocarbon receptor (AhR), constitutive androstane receptor (CAR) and pregnane X receptor (PXR), respectively. Messenger RNA (mRNA) expression of AhR, CAR and PXR and the activity of AhR were upregulated after AFs treatment in hepatic cell lines (Ayed-Boussema et al., 2012; Mary et al., 2015; Vogel et al., 2020). These data provide a foundation for the CYPs regulation pathway to explain how AFs enhance the expression of CYPs enzymes. Notably, AFs may have a planar polycyclic aromatic structure similar to known ligands that allows AFs to induce the subsequent transcription of target genes via binding to receptors (Mary et al., 2015). Because of the highly reactive and electrophilic natures of the metabolic intermediates produced by microsomal CYPs, these molecules covalently bind to proteins and DNA to exert acute and chronic cytotoxicities.

Glutathione-S-transferases (GSTs) are responsible for detoxification of the AFs, which can catalyze nucleophilic aromatic substitutions, Michael additions to α,β-unsaturated ketones and epoxide ring-opening reactions, resulting in the formation of glutathione (GSH, a cysteine-containing tripeptide) conjugates (Jowsey et al., 2003) and oxidized glutathione (glutathione disulfide, GSSG) (Sheehan et al., 2001; Eftekhari et al., 2018). By reacting with reactive oxygen species such as free radicals or peroxides, GSH serves as an electron donor and is converted to its oxidized form as GSSG, and vice versa, GSSG can be reduced back to GSH by glutathione reductase (GR). The GSH and GSSG pair, as one of the major intracellular redox regulating couples, plays an important role in protecting cells from oxidative stress that is caused by imbalance between pro-oxidants and antioxidants, for which depletion of GSH and accumulation of GSSG suggest the toxic effects of AFs on the liver cells (Adeleye et al., 2014; Ali Rajput et al., 2017). Conjugation of GSH with metabolites of AFs results in the generation of more soluble compounds that are more easily detoxified and excreted from cells. Upon further metabolism, these thiol conjugates are excreted in urine as water-soluble aflatoxin mercapturic acids (AFB_1_-NAC) (Kensler et al., 2011, Kenseler et al., 2014). When we focus on the hepatic toxicity of AFs, it is important to note that the toxicity of AFs depends on the type, dose, duration of exposure and the susceptibility of different species. In experimental models, mice possess a much higher level of hepatic GST activity toward AFBO than found in rats or humans (Taguchi et al., 2016). Newborn mice, which have lower hepatic GST levels than older mice, are substantially more susceptible to AFs (Kensler et al., 2014). These peculiarities suggest that the selection of doses and models is important during the research on AFs-induced hepatotoxicity. In addition, in almost all of researches, there are similar laboratory findings about AFs-induced hepatotoxicity. And many researches prefer using younger experimental models that are more susceptible to AFs exposure like one-day-old broilers for a more obvious phenomenon (Mughal et al., 2017; Muhammad et al., 2018b; Wang et al., 2018). But it’s worth thinking whether the different ages of experimental models will lead to different findings in related mechanisms of AFs-induced hepatotoxicity, such as showed in 7.1.2. And Li Huang et al. found that 300 μg/kg was a appropriate dose for male ICR mice, showing a moderate toxicity symptoms in the pre-experiment. Because humans usually expose to AFs chronically in a very low dose, in order to simulate the actual situation more realistically, a consideration of AFs exposure dose is needed in the future.

## AFs Damage Mitochondria to Initiate Various Cellular Injury Pathways

### AFs Induce the Destruction and Dysfunction of Mitochondria

Mitochondria in hepatocytes consume approximately 90% oxygen and undertake the important function of oxidative phosphorylation (OXPHOS). At the presence of activated metabolites of AFs after bioactivation, AFs destroy the mitochondrial respiratory chain complexes I-IV, which regulate the delivery of electrons and produce adenosine triphosphate (ATP) for cell energy consumption (Shi et al., 2012a). AFs reduce the activity of respiratory chain complexes, which leads to a decrease in ATP synthesis. This finding is consistent with the decrease in the mitochondrial respiratory control ratio (RCR) (Shi et al., 2012a). The AFs-induced decrease in the activities of succinate dehydrogenase (SDH) and adenosine triphosphatase (ATPase) also support mictochondrial dysfunction (Verma et al., 2008). AFs also disrupt lipid and lipoprotein metabolism. For example, AFs downregulate carnitine palmitoyl transferase 1 A (CPT1A) in a dose-dependent manner, which aids long chain fatty transport into the mitochondria for *β*-oxidation (Rotimi et al., 2017). Related serum and histological examinations also confirmed the disorder of lipid metabolism (Alm-Eldeen et al., 2015; A V et al., 2017).

AFs also destroy the intact mitochondrial bilayer structure via regulating B cell lymphoma2 (Bcl-2) family proteins. Bcl-2 exerts its anti-apoptotic activity via binding of the activated Bax effector to prevent mitochondrial outer membrane permeabilization (MOMP) in healthy hepatocytes. However, decreased Bcl-2 protein levels were found in hepatocytes exposed to AFs (Liao et al., 2014; Chen et al., 2016; Alm-Eldeen et al., 2017; Bock and Tait, 2020). Bax dimers form higher-order multimers that generate lipid pores within the outer mitochondrial membrane (OMM), which causes MOMP and the release of intermembrane space contents, such as cytochrome c (cyt c). AFs increased Bax expression in many studies (Liao et al., 2014; Oskoueian et al., 2015; Chen et al., 2016; Liu and Wang, 2016; Alm-Eldeen et al., 2017; Wang et al., 2018,^2019^; Huang et al., 2019; Deng et al., 2020). Strikingly, the translocation of Bax was observed, and Bax levels increased in the mitochondrial fraction (Chen et al., 2016; Liu and Wang, 2016). A decreased ratio of Bcl-2/Bax, as an apoptotic index, was demonstrated after exposure to AFs (Liao et al., 2014; Alm-Eldeen et al., 2017). The increase in p53 tumor suppressor proteins also participated in the destruction of mitochondria via modulation of the balance between Bcl-2 and Bax (Liao et al., 2014). AFs induced p53 expression in the nucleus, which contributes to mitochonria-induced apoptosis (Sun et al., 2015a; Wang et al., 2019). Besides, AFB_1_ or AFM_1_ activate c-Jun N-terminal kinase (JNK), which modulates the mitochondrial ratio of Bcl-2/Bax proteins (Zheng et al., 2018). Mitochondria, the major organelle of liver, are vulnerably attacked by AFs-induced dysfunction and apoptosis because of its involvement in ROS production and oxygen consumption in cells. As the initial event of intrinsic apoptosis, the persistent opening of mitochondrial permeability transition pore (mPTP) contributes to dysfunction of mitochondria after AFs exposure through inducing collapse of mitochondrial membrane potential (MMP) and making mitochondria swell, and subsequently produces osmotic dysregulation of the inner mitochondrial membrane, uncoupling of oxidative phosphorylation and cell death.

Mitochondria-centered cell death also disrupts the membrane structure of liver cells, which causes the release of some cell contents. The leakage of ALT from the cytoplasm and AST from mitochondria into serum was reported in many studies (Akçam et al., 2013; Liao et al., 2014; Alm-Eldeen et al., 2017; Vipin et al., 2017; Huang et al., 2019). Histological and ultrastructural analyses also confirmed 1) swollen, fragmented and damaged mitochondria, 2) irregular and condensed nuclei with fragmentation and clustering of chromatin material, and 3) the formation of horseshoe or crescent-shaped bodies and ultimate cell death of hepatocytes (Yang et al., 2016; Mughal et al., 2017; Muhammad et al., 2018b; Wang et al., 2018).

### Reactive Metabolites of AFs Influence Mitochondria via Binding With Mitochondrial DNA

Mitochondria also maintain their own genome, which encodes the OXPHOS functional proteins. Mitochondrial DNA (mtDNA) is highly susceptible to oxidative stress due to its proximity to the inner membrane (a primary source of reactive oxygen species (ROS)) and the less protection of histones (Begriche et al., 2011; Shi et al., 2012). Sequence analysis of the mtDNA D-loop indicates that AFs induce a few single nucleotide polymorphisms (SNPs) in the mtDNA D-loop, which interfere with transcription of the entire mtDNA genome (Shi et al., 2015). The comet assay, which measures cellular DNA repair capacity and DNA damage, indicates that ROS, including the reactive metabolites of AFs and other factors cause the breaking of DNA strand including mtDNA (Yang et al., 2016; Vogel et al., 2020). At the same time, an oxidized nucleoside of DNA, 8-hydroxy-2′-deoxyguanosine (8-OHdG) is may be one of the predominant forms of free radical-induced oxidative lesions, and its content increased after AFs treatment (Valavanidis et al., 2009; Lin et al., 2016; Yang et al., 2016). DNA fragmentation analysis demonstrates that DNA fragments were increased in a dose-dependent manner (Chen et al., 2016). These data suggest that AFs influence the coding of respiratory chain complex proteins and the dysfunction of mitochondria by impairing mtDNA because OXPHOS requires the mtDNA to encode 13 mitochondrial respiratory chain (MRC) polypeptides, which are embedded with complexes I, III, IV, and V (Begriche et al., 2011). However, more concrete evidence must be substantiated further about how AFs exactly influence mitochondria after binding with mtDNA and preventing it from functioning properly.

### AFs Induce Extensive Oxidative Stress in Mitochondria

Some electrons from complexes I and III also directly react with oxygen under normal physiological conditions to form the superoxide anion radicals. What’s worse is that AFs stimulate mitochondria to generate MOMP and damage respiratory complexes, which leads to the release of electrons from MRCs in force. Superoxide anion radicals in rat hepatocytes treated with AFB_1_ increased significantly (Ajiboye et al., 2016). Mitochondrial manganese superoxide dismutase (Mn-SOD) is the committed enzyme of the antioxidant pathway that catalyzes radicals, such as O_2_^−^, into the relatively less toxic hydrogen peroxide (H_2_O_2_), which is catalyzed by glutathione peroxidase (GPx) and catalyzed into water by GSH (Begriche et al., 2011; Pessayre et al., 2012; Maurya and Trigun, 2016). AFs downregulated the expression and activity of these enzymes, which amplified oxidative stress (Vipin et al., 2017; Gan et al., 2018). The decreasing content of GSH as a cofactor or coenzyme that is involved in the enzymatic detoxification of ROS derived by AFs is an adverse effect for maintaining the redox homeostasis. Some of the formed H_2_0_2_ that leaks from the mitochondria into the cytoplasm is split into hydroxyl radicals (OH·) and hydroxide (OH-) at the presence of iron. The reaction called Fenton reaction also contributes to the formation of ROS and takes part in oxidative stress (Pessayre et al., 2012), but there have been no reports about the role of the Fenton reaction in AFs-induced oxidative stress yet.

Damaged mitochondria result in the generation of ROS. AFs-treated hepatocytes generated high levels of ROS in numerous studies (Liu and Wang, 2016; Yang et al., 2016; Vipin et al., 2017; Eftekhari et al., 2018; Xu et al., 2020). This finding is consistent with the increased content of a series of lipid peroxidation products in AFs-treated hepatocytes, e.g., conjugated dienes, lipid hydroperoxides and malonaldehyde (MDA). And MDA, a terminal marker of lipid peroxidation, is an important factor that reflects alteration of membrane fluidity (Gesing and Karbownik-Lewinska, 2008; Shi et al., 2012b; Ajiboye et al., 2016; Erdélyi et al., 2018). Protein oxidation or carbonylation are important oxidative steps of toxicities in mitochondria. For instance, at the presence of oxygen free radicals, increased inducible nitric oxide synthase (iNOS) that mediates the catalytic formation of nitric oxide, induces the formation of oxidative protein biomarkers of nitrotyrosine. In addition, 8-OHdG as an oxidative DNA biomarker is obviously increased during AFs-induced mitochondrial oxidative stress (Ozen et al., 2009; Karaman et al., 2010). According to the literature, AFB_1_ may provoke earlier formation of ROS, subsequently affect on gene expression with the cascade of inhibition of Kelch-like ECH-associated protein 1 (Keap1) and activation of nuclear factor erythroid 2-related factor 2 (Nrf2)/antioxidant response element (ARE) pathway, then accompanied with activation of the antioxidant gene cluster of GPx4 to prevent the completion of lipid peroxidation processes (Kövesi et al., 2018). Lipid peroxidation alters membrane fluidity and permeability. And lipid peroxides are also toxic for hepatocytes (Gaschler and Stockwell, 2017). Meanwhile, 4-hydroxylnonenal, as a major end product from lipid peroxidation during oxidative stress, may enhance gene expression of cyclooxygenase (COX)-2 in rat liver epithelial RL34 cells, of which is highly reactive to nucleophilic sites in DNA and proteins, causing cytotoxicity and genotoxicity (Kumagai et al., 2000).

## AFs Influence the Cell Cycle and Growth via Post-Transcriptional Regulation of miRNAs

MiRNAs are a class of small non-coding RNA molecules that regulate gene expression via base-pairing with complementary sequences within mRNAs, generally with the 3′ untranslated region (3′-UTR) of miRNAs (Fang et al., 2013; Sanjay and Girish, 2017). AFs upregulated many miRNAs, such as miR-34a-5p, miR-33a-5p, and miR-122–5p in different experimental models (Fang et al., 2013; Yang et al., 2014; Liu et al., 2015; Zhu et al., 2015; Marrone et al., 2016; Livingstone et al., 2017). Genome-wide miRNA profiling and Kyoto Encyclopedia of Genes and Genomes (KEGG) pathway analysis provide evidences that AFs regulate potential target genes of differentially expressed miRNAs involved in the p53 signaling pathway, cell cycle, Wnt signaling pathway and Janus kinase (JAK)/signal transducer and activator of transcription (STAT) signaling pathway (Yang et al., 2014). Upregulation of miR-34a-5p suppressed the expression of the cell cycle-related genes, such as cyclin D1 (CCDN1) and cyclin E2 (CCNE2), which led to cell cycle arrest in the G0-G1 phase. A tight connection between miR-34a-5p and p53 was found and validated in a p53 small interfering RNA (siRNA) knockdown experiment (Liu et al., 2015). Yi Fang et al. found that AFs upregulated the level of miR-33a *in vitro*. The luciferase assay found that miR-33a downregulated *β*-catenin to inhibit cell growth via direct binding to the 3′-UTR of *β*-catenin. Additionally, the upregulation of miR-34a by AFs also inhibited the Wnt/β-catenin pathway via *β*-catenin. Anti-miR-34a significantly relieved the downregulated *β*-catenin and its downstream genes, such as c-myc and cyclin D1, and AFs-induced cell cycle arrest was also relieved in HepG2 cells (Zhu et al., 2015). AFs also downregulated the expression of miR-130a and miR-122 (Liu et al., 2015; Marrone et al., 2016). The Downregulation of Drosha, DiGeorge syndrome critical region 8 (DGCR8) and Dicer, which are enzymes in the maturation of miRNAs, indicates that AFs induce an impairment of miRNA biogenesis (Zhu et al., 2015). AFs down-regulated miR-122 via inhibition of hepatocyte nuclear factor 4 A (HNF4A), which regulates the expression of miR-122 (Marrone et al., 2016).

## AFs Induce Programmed Cell Death to Cause Hepatotoxicity

### Intrinsic Apoptosis Pathway (Mitochondrial Pathway)

When AFs-induced damage on mitochondria and normal structures of hepatocytes exceeds the cellular repair capacity such as antioxidant system and autophgy, hepatocytes initiate apoptosis to maintain normal liver function, but also cause hepatotoxicity. Apoptosis, also called programmed cell death (PCD), has two main routes, the intrinsic and extrinsic pathways, to induce hepatotoxicity via the activation of caspase (cysteinyl, aspartate-specific protease), which leads to the cleavage of multiple intracellular substrates (Chalah and Khosravi-Far, 2008; Brenner and Mak, 2009). The opening of the mPTP is the most well-documented and important initiating event. According to existing findings, after AFs exposure, AFs are activated metabolically to react with proteins and DNA, which can damage mitochondria and induce metabolic inhibition, initiating the opening of mPTP. The appearance of swollen mitochondria suggestes that AFs open the mPTP to initiate the apoptosis (Essa et al., 2017; Baines and Gutiérrez-Aguilar, 2018; Briston et al., 2019). MMP analysis also demonstrates that AFs induce the collapse of MMP, which results from the opening of mPTP (Liu and Wang, 2016).

The AFs-induced opening of mPTP releases electrons, cyt c (the key in the mitochondria-dependent apoptosis pathway) and molecules less than 1,500 Da (Chen et al., 2016; Yang et al., 2016; Eftekhari et al., 2018). As mentioned before, AFs also damage mitochondria via regulation of Bcl-2 family proteins to leak out contents. The leakage of cyt c was found in the AFs-induced mitochondria-mediated apoptosis (Chen et al., 2016; Wang et al., 2018). Immunoprecipitation analysis indicates that AFs-induced cyt c release from mitochondria into the cytosol constitutes the apoptosome complex, which includes apoptotic proteases activating factor-1 (Apaf-1), cyt c and caspase-9 (Chen et al., 2016). AFs also increased the level of caspase-9 at the same time (Liu and Wang, 2016; Yang et al., 2016; Mughal et al., 2017; Wang et al., 2018; Xu et al., 2020). The downstream product of the caspase cascade, caspase-3, is an executioner that degrades intracellular structural and functional proteins, and it was also increased (Liao et al., 2014; Chen et al., 2016; Liu and Wang, 2016; Yang et al., 2016; Mughal et al., 2017; Wang et al., 2018; Huang et al., 2019; Xu et al., 2020). The increased number of apoptotic cells also confirmed these consequences using flow cytometry analysis (Liu and Wang, 2016; Mughal et al., 2017).

### Extrinsic Apoptosis Pathway

The extrinsic cell death pathway is typically triggered by death receptor-ligand interactions, which are important initiators of apoptosis. Death receptors recognize cytokines in the liver, including Fas, tumor necrosis factor receptor 1 (TNFR1), and tumor necrosis factor-related apoptosis inducing ligand receptors 1 and 2 (TRAIL-R1 and TRAIL-R2) (Malhi et al., 2006). The significantly increased expression of these receptors, such as TNFR1 and Fas, indicates that AFs invoke apoptosis via the death receptor pathway in hepatocytes. TNFR1 comprises of TNFR-associated protein with death domain (TRADD), TNF-associated factor-2 (TRAF2) and receptor-interacting protein (RIP). AFs increase the expression of TRADD and TRAF2 (Mughal et al., 2017; Huang et al., 2019). The recruitment of the adaptor protein Fas-associated protein with death domain (FADD) also increased in hepatocytes treated with AFs (Mughal et al., 2017; Huang et al., 2019). After the oligomerization and activation of receptors, all three classes of receptors initiate the cleavage of pro-caspase-8 and/or -10 to activate caspase-8/10, which directly activate downstream caspases, such as caspase-3/6/7 (Malhi et al., 2006). The expression of caspases-8 and -10 increased as expected (Mughal et al., 2017). The livers of rats that received AFs in their feed had positive expression of caspase-3 in the cytoplasm of some apoptotic cells and apoptotic bodies (Essa et al., 2017). Another pattern of cell death, necrotic parenchymal hepatocytes, which are characterized by cytoplasmic vacuolar degeneration, were also observed (Alm-Eldeen et al., 2015). The end point of the intrinsic and extrinsic pathways is the activation of intracellular proteases and endonucleases that eventually degrades the cellular constituents (Malhi et al., 2006). In conclusion, AFs destroy the function and structure of hepatocytes to induce cell death via various pathways, and inhibit cellular protective processes to further aggravate hepatotoxicity.

## AFs Aggravate Hepatotoxicity via the Destruction of Survival Pathways

### AFs Affect Autophagy to Inhibit the Cellular Protective Process

#### AFs Block the Process of Autophagy

Autophagy (macroautophagy) contributes to cell survival via the transfer of damaged organelles, such as mitochondria damaged by AFs, and proteins into lysosomes for the recycling of subcellular debris and metabolic intermediates to maintain normal cell homeostasis (Muhammad et al., 2018b). During autophagy, an isolated double membrane structure called an autophagosome encloses some cytoplasmic materials, including damaged organelles and unused proteins. The outer membrane of the autophagosome fuses with the membrane of lysosomes to constitute autolysosomes that degrade isolated components via lysosomal enzymes. Ultra-microstructural observation of hepatocytes in an AFs-treated group found few autophagosomes. This result suggests that AFs inhibit the process of autophagy (Wang et al., 2019).

Multiple autophagy-related (Atg) proteins regulate autophagosome initiation and maturation. Upon the introduction of autophagy, a preinitiation complex translocates to the pre-autophagosomal structure (PAS). The preinitiation complex recruits and activates an autophagy-specific form of the phosphoinositide 3-kinase (PI3K) complex, which includes Beclin-1/Atg6, Vps34, Vps15 and Atg14 to the PAS. Notably, AFs decreased the mRNA expression of Beclin-1 (Muhammad et al., 2018b; Wang et al., 2019). However, most articles focused on the formed autophagosome, and more exact evidence of the introduction of autophagosomes must be provided to delineate the effects of AFs on autophagic hepatocytes in the beginning.

**FIGURE 2:**
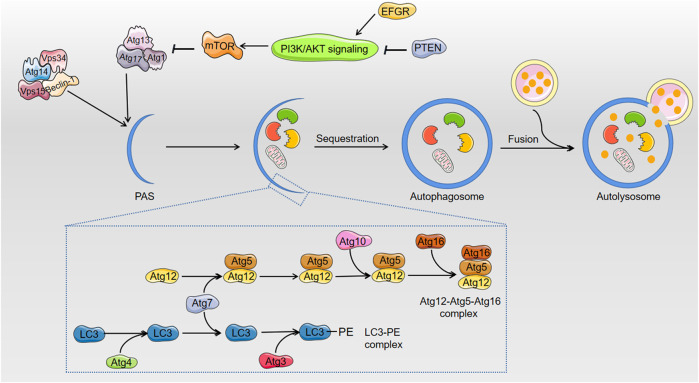
The related regulation of autophagy after exposure to AFs. AFs block up the process of cell survival pathway: autophagy. In the course of elongation and sequestration of autophagosome, AFs inhibit autophagy through decreasing the expression of Beclin-1, Atg5, and LC3. As to the regulation of autophagy, AFs can down-regulate the expression of mTOR to indirectly inhibit autophagy. AFs also indirectly inhibit autophagy via EFGR that regulates mTOR pathway.

Figure 2 shows that the elongation and enclosure of the autophagosome during the sequestration process of autophagy require two protein conjugation systems, the Atg12-Atg5-Atg16 complex and light chain 3 (LC3)/Atg8-phosphatidylethanolamine (PE) complex, respectively (Green and Llambi, 2015; Lin and Baehrecke, 2015). AFs inhibit autophagy via blocking the expression of the components of the two complexes. For example, AFs downregulated the mRNA expression of Atg5 (Muhammad et al., 2018b). The inhibition of AFs on autophagy was confirmed via detecting the distribution of GFP-LC3 (a sort of green fluorescent protein inserted into LC3) puncta, which represent autophagosomes (Xu et al., 2020). LC3-II plays an indispensable role in autophagosome formation, and the LC3-II/LC3-I ratio is used as the most dependable biomarker of autophagy. The decreasing protein level of LC3 and the reduced LC3-II/LC3-I ratio also indicated that AFs inhibited the cell protective process of autophagy (Muhammad et al., 2018b; Wang et al., 2019). Mammalian target of rapamycin (mTOR), which is a protein kinase, negatively regulates autophagy via phosphorylation of ULK1 and Atg13 to decrease the affinity of Atg13 with ULK1 and the activity of ULK1 (Green and Llambi, 2015). AFs exposure increased mRNA and protein expression of mTOR, which supports that AFs inhibit autophagy via various mechanisms (Muhammad et al., 2018b; Wang et al., 2019; Xu et al., 2020). AFs also indirectly inhibit autophagy via regulation of epidermal growth factor receptor (EGFR), which regulates the mTOR pathway as a direct upstream kinase of PI3K/AKT (Xu et al., 2020).

On basis of AFs-induced mitochondrial damage, for selective autophagy, mitophagy is the sequestration of damaged mitochondria by autophagosomes, which display loss of membrane potential. Both *in vitro* and *in vivo* studied indicate that AFB_1_ promotes mitophagy by increased Parkin and PTEN-induced putative kinase 1 (PINK1) through inducing the expression of COX-2, then results in lipid accumulation and steatosis, due to the fact that mitophagy disrupts the normal mitochondrial lipid metabolism function (Ren et al., 2020). But in consideration of positive role of mitophagy in removing damaged mitochondria and maintaining its quality and metabolism, more explicit evidences about AFs-induced mitophagy in hepatotoxicity need to be excavated.

#### The Dual Role of AFs in the Regulation of Autophagy

Do AFs invariably inhibit the process of autophagy? The answer is not necessarily yes. Different results from a previous paper showed that AFB_2_ increases the level of autophagy. Fluorescent antibodies against LC3, MDC staining and transmission electron microscopy (TEM), show that AFB_2_ induce autophagy. AFB_2_ also increases the ratio of LC3-II/I and Beclin-1. A decrease in p62, which is an autophagic flux marker in the protein degradation pathway, is also found. AFB_2_ also activate PI3K, Akt and mTOR and inhibit their phosphorylation to inhibit the PI3K/Akt/mTOR pathway, which shows the proautophagic activity of AFB_2_ (Chen et al., 2016). Table 1 shows that the lower dose of AFs and older experimental models may be the cause of the different results. However, this conjecture needs to be confirmed in the future.

**TABLE 1:** Effect of different types and doses of AFs and experimental models on autophagy.

References	Experimental model	The age of experimental animal models	The dose of AFs	The type of AFs	Effect on autophagy
Zhu et al. (2015)	Broiler chickens	1-day-old	5 mg/kg	AFB_1_	Inhibit
Liu et al. (2016)	Human L02 cells	–	40 μM	AFB_1_	Inhibit
Verheecke et al. (2016)	Broilers	121-day-old	0.2/0.4/0.8 mg/kg	AFB_2_	Promote
Zheng et al. (2018)	Arbor acres broilers	1-day-old	5 mg/kg	AFB_1_	Inhibit

The lengthy autophagy and intricate cellular environment decide that autophagy will be influenced by many proteins and cell processes. One report showed that AFs-induced autophagy and apoptosis exhibit a reciprocal inhibition (Xu et al., 2020). In addition, most of the literatures focused on the formation of autophagosomes, and the questions of fusion with lysosomes persist.

### The Collapse of the Antioxidant System Keap/Nrf2/ARE Pathway

Under the stress of the constantly generated ROS and reactive metabolites of exogenous mycotoxin AFs, the Keap1/Nrf2/ARE pathway plays an essential role in maintaining cellular redox homeostasis and protecting hepatocytes from the generation of cascade reactions induced by ROS and electrophilic agents (Jowsey et al., 2003; Tu et al., 2019). Under normal physiological conditions, Nrf2 is ubiquitinated via binding to Keap1, which leads to the degradation of Nrf2, so Nrf2 activation is constantly maintained at a low levels. When hepatocytes encounter AFs-induced oxidative stress, modification of Keap1 stabilizes Nrf2, which accumulates in the nucleus where it binds to ARE in conjunction with small Maf proteins to induce the expression of related antioxidant genes (Taguchi et al., 2016). However, it has found that AFs downregulated the expression of Nrf2 (Vipin et al., 2017; Muhammad et al., 2018a; Muhammad et al., 2018b; Li et al., 2019; Rajput et al., 2019). AFs regulate Nrf2 not only via Keap1 but another protein-Caveolin-1 (Cav-1) (Bryan et al., 2013). Cav-1 is a major resident scaffolding protein constituent of caveolae, which recruit numerous signaling molecules to caveolae and regulate their activities (Cohen et al., 2004). AFs upregulate the expression of the Nrf2 inhibitor Cav-1 to suppress its activity in hepatocytes, which results in the attenuation of cellular antioxidant capability (Xu et al., 2020).

The Keap1/Nrf2/ARE pathway regulates the expression of many antioxidant enzymes and phase II detoxifying enzymes, such as heme oxygenase-1 (HO-1), GR, glutathione peroxidase (GSH-Px), NAD(P)H dehydrogenase, quinone 1 (NQO1), GST, and SOD. AFs downregulated many antioxidants, including SOD, HO-1, NQO1, GSH-Px, catalase (CAT), GST, and GR, in many studies and experimental models (Yener et al., 2009; Alm-Eldeen et al., 2015; Yang et al., 2016; Vipin et al., 2017; Muhammad et al., 2018a; Muhammad et al., 2018b; Ji et al., 2020). However, one study found that AFs upregulated the expression of Nrf2 (Liu and Wang, 2016).

AFs-induced dysfunction of the Nrf2/ARE antioxidant system, which is one of the dominant cellular protective mechanisms, will magnify the oxidant stress due to the effects of AFs and their reactive metabolites (Abdel-Aziem et al., 2014; Sun et al., 2015b; Qin et al., 2016). These studies show an important role of the Keap1/Nrf2/ARE antioxidant response element and its downstream enzymes in the detoxification and metabolism of AFs. In short, AFs and their reactive metabolites cause damage directly, and profoundly influence liver cells via downregulation of the Keap1/Nrf2/ARE antioxidant system.

## AFs Amplify Hepatotoxicity in the Shape of a Vice Feedback Loop--Inflammation

### AFs Regulate the NF-κB Signaling Pathway to Initiate Inflammation

Inflammation is a defense mechanism that obliterates harmful substances when the body is under attack by xenobiotics, such as AFs, and bacterial lipopolysaccharide (LPS) and endogenous substances, such as damage-associated molecular patterns (DAMPs), which are released from AFs-induced damaged hepatocytes. Although inflammation is essential for the initiation of protective immunity, unregulated inflammation causes tissue destruction and the development of inflammatory diseases (Afonina et al., 2017). Abundant experimental evidence from examinations of liver sections indicate that inflammation occurs in AFs-treated groups (Yener et al., 2009; Vipin et al., 2017; Muhammad et al., 2018a). When AFs start to destroy the structures and normal physiological functions of hepatocytes, these cells initiate inflammation via the two canonical molecular drivers of the inflammatory response, the transcription factor nuclear factor-kappaB (NF-κB) and Nod-like receptor pyrin domain containing 3 (NLRP3) inflammasome (Kubes and Mehal, 2012; Afonina et al., 2017).

Some different receptors trigger pro-inflammatory NF-κB signaling pathways, including TNFR1, interleukin (IL)-1R and Toll-like receptor (TLR) (Kubes and Mehal, 2012; Afonina et al., 2017). Unsurprisingly, the upregulation of TLR-2/4 was verified at different doses of AFs (Muhammad et al., 2018b; Huang et al., 2019; Wang et al., 2019). Several papers found that the mRNA and protein expression of NF-κB was upregulated in hepatocytes after exposure to AFs (Ma et al., 2015; Oskoueian et al., 2015; Muhammad et al., 2018b; Chen et al., 2019a; Huang et al., 2019; Zhang et al., 2019). NF-κB binds to an inhibitory protein called IκB in the resting state and remains sequestered in the cytoplasm. Although upstream signaling events differ between individual receptors, the IκB-kinase (IKK) complex is eventually activated, which subsequently inactivates the inhibitory chaperone IκBα of NF-κB (Afonina et al., 2017). Several research papers demonstrated that the expression level of IκB was inhibited in AFs group (Chen et al., 2019a; Huang et al., 2019). The mRNA level of IKK increased, but it was not significant (Mughal et al., 2017). Therefore, the role of the IKK complex in the regulation of NF-κB after exposure to AFs remains to be validated.

Once IκB is degraded, free NF-κB dimer translocates into the nucleus, which regulates the expression of inflammatory cytokines, such as IL-1 and TNF family members. Notably, AFs induced higher NF-κB p65 expression in the nucleus than in the cytoplasm (Huang et al., 2019; Wang et al., 2019; Zhang et al., 2019). The expression of pro-inflammatory cytokines, including TNF-α, IL-1, IL-1β, IL-6 and IL-8, was upregulated at the transcriptional and serous levels (Ma et al., 2015; Muhammad et al., 2018b; Chen et al., 2019a; Taranu et al., 2019; Wang et al., 2019; Owumi et al., 2020). All of these histological observations and data of the NF-κB-related signaling pathway indicate that NF-κB plays an essential role in AFs-induced hepatic inflammation infiltration. However, it is critical that the activation of caspase-1 and IL-1β contributes to the inflammatory process via the molecular complex known as the inflammasome (Qin et al., 2016).

### AFs Regulate Inflammasome Assembly and Inflammation

The NLRP3 assembly forms a large cytosolic protein complex, the NLRP3 inflammasome, as a molecular platform that results in the self-cleavage and activation of cysteine protease caspase-1, which converts pro-IL-1β and pro-IL-18 into their mature forms (Afonina et al., 2017). NLRP3 inflammasome activation requires two sequential steps, initiation and activation. Initiation entails the recognition of DAMPs by TLR, which results in the activation of NF-κB, induction of pro-IL-1β and increased synthesis of NLRP3. During the activation step, diverse extracellular stimuli trigger the assembly of the inflammasome complex and its eventual activation (Woolbright and Jaeschke, 2017).

Activated caspase-1 also forms a pore in the cellular plasma membrane via the cleaving of gasdermin D, and caspase-1-dependent pyroptosis maintains inflammation via the release of DAMPs from dying hepatocytes (Yang et al., 2014). The levels of proteins that represent assembly of the NLRP3 inflammasome, such as NLRP3, caspase recruitment domain (ASC) and p10 (the active form of caspase 1), and symbolize activation, such as IL-1β and GSDMD, in liver tissue were enhanced in AFs-exposed mice, and the features of pyroptosis, which are characterized by pyroptosis bodies in the early stage and membrane rupture in the terminal stage, were observed. Notably, dephosphorylation of COX-2 plays a role in the activation of the NLRP3 inflammasome via protein phosphatase 2 A (PP2A)-B55δ induced by AFs and alters its ER localization and ER-associated degradation (ERAD) (Ou et al., 2014; Zhang et al., 2019).

There is evidence that mitochondria are also key intermediate that induce NLRP3 activation via AFs-induced mitochondrial damage and the subsequently released contents, such as mtDNA and ROS (Kubes and Mehal, 2012; Afonina et al., 2017). Prior evidences that damaged AFs-induced mitochondrial damage lay a foundation in the area of inflammasome activation. Notably, increased mitochondrial translocation of NLRP3 and its colocalization with ASC suggest that mitochondria play a role in the recruitment and activation of the NLRP3 inflammasome in AFs-induced liver injury (Zhang et al., 2019). However, more evidence should shed light on more specific mitochondrial functions in the regulation of the NLRP3 inflammasome to induce pyroptosis and inflammation. NLRP3 inflammasome binds adjacent NLRP3 monomers and licenses self-nucleation through

formation of an 11 or 12-subunit disc-like complex (Alehashemi and Goldbach-Mansky, 2020), which assembles ASC and caspase-1 filaments. NLRP3 can be activated by mitotic kinase at the leucine-rich repeat (LRR) domain. In contrast to NLRP3, NLRP1 is expressed not only in immune cells and tissues but non-hematopoietic tissues, which has a FIIND domain, a pyrin domain (PYD) and a caspase activating recruitment domain (CARD) domain and can activate caspase-1 independent of the adapter ASC. NLRP1 is a sensor for proteases and pathogen effectors, that can directly induce proteasomal degradation of NLRP1 (Dowling and O'Neill, 2012). Both NLRP3 and NLRP1 are associated with inflammation related cascade, whose oligomerization are inhibited by high K^+^ concentrations, revealed differences in assembly, and their downstream function. Two classic inflammatory pathways, the NF-κB and NLRP3 inflammasomes, contribute to the initiation of the inflammation response when the liver is under the attack of AFs. Once inflammation is started, the local hepatic immune microenvironment determines the extent of liver damage via macrophages to induce secondary cell death.

### AFs Exacerbate the Local Hepatic Inflammatory Microenvironment via Macrophages

Liver macrophages are the primary liver-resident phagocytes also known as Kupffer cells (KCs) and bone marrow-derived recruited monocytes. Histological examination demonstrated that mononuclear cell and lymphocyte infiltration occurred in AFs-induced hepatotoxicity (Yener et al., 2009; Shi et al., 2015; Vipin et al., 2017). Among the liver macrophages, KCs are located on the luminal side of the hepatic sinusoidal endothelium (Krenkel and Tacke, 2017; Guillot and Tacke, 2019). The physiological features of sinusoidal cells determine the importance of KCs in sensing the microenvironment via long cytoplasmic expansions after exposure to AFs.

As described earlier, AFs damage hepatocytes to release DAMPs and pro-inflammatory cytokines, including IL and TNF, to activate KCs, which causes transcriptional activation of cytokine formation and activation of the inflammasome (Singh et al., 2015; Woolbright and Jaeschke, 2017; Zhang et al., 2019). AFs induce M1-like polarization to cause liver damage (Tacke and Zimmermann, 2014; An et al., 2017). The higher content of iNOS and NO in the liver also demonstrated that AFs induce the M1 polarization of macrophages to promote inflammation (El-Nekeety et al., 2014; Li et al., 2014; Ajiboye et al., 2016). The addition of a derivative of the methylxanthine theobromine as an inhibitor of TNF-α alleviated the toxic effect of AFs in an isolated perfused liver model (Koohi Mohammad et al., 2017). The Use of Transwell chamber for co-culture to simulate the interaction between KCs and hepatocytes significantly upregulated NLRP3, ASC, IL-1β and GSDMD proteins (Zhang et al., 2019). These data indicate that KCs form a negative feedback loop to enhance liver inflammation injury under the stimulation of AFs. However, whether the role of macrophages is definitely negative is not known. Innate immune cells also use macrophage extracellular trap (MET), which is a reticulate structure based on extracellular DNA, to degrade the exogenous AFs and weaken their toxicity (An et al., 2017). In summary, AFs induce the release of cytokines and DAMPs via activation of NF-κB and inflammasome pathways in hepatocytes and macrophages to form a local inflammatory microenvironment and negative feedback loop.

## Conclusion and Perspective

**FIGURE 3:**
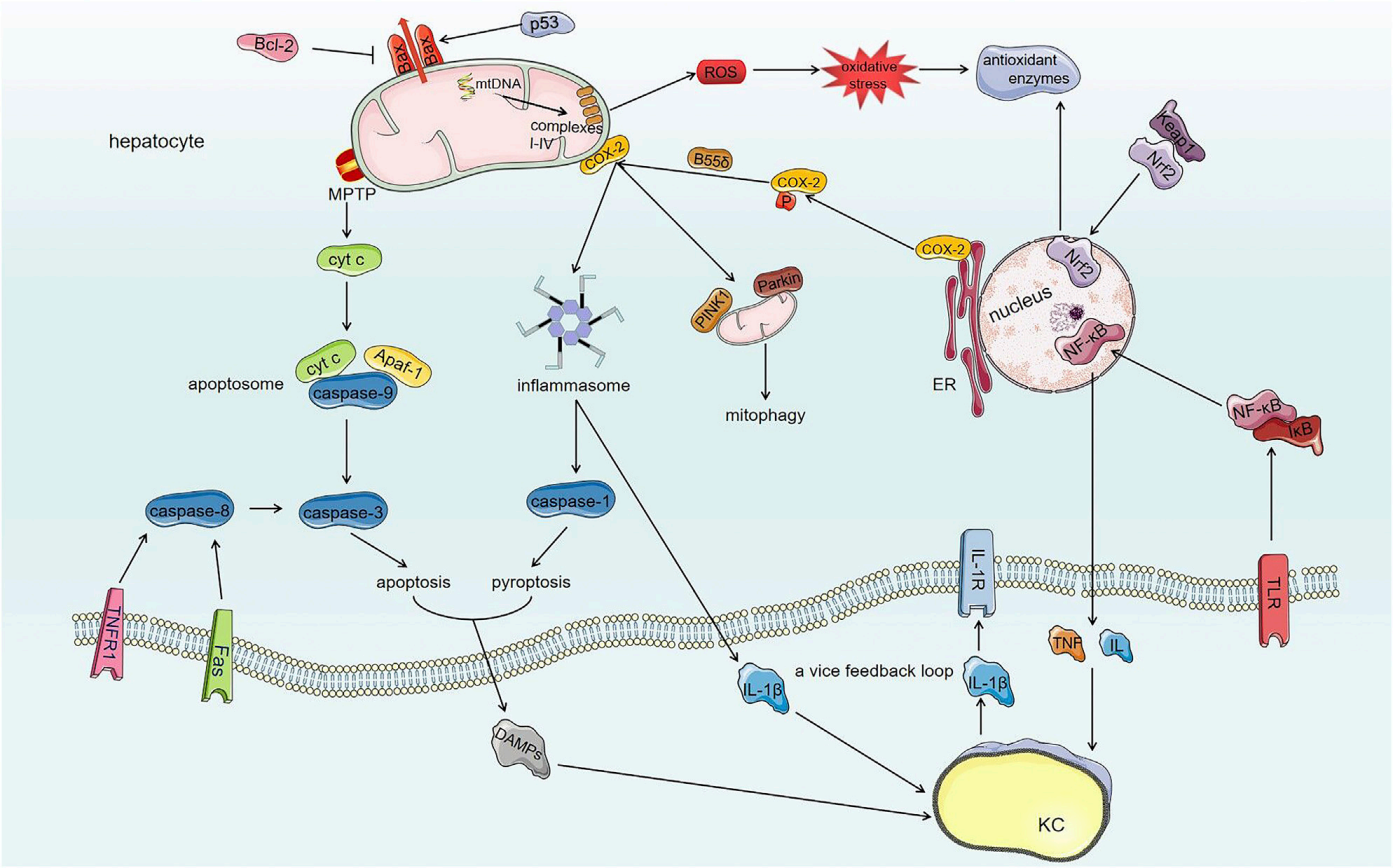
The toxic mechanisms of AFs-induced hepatotoxicity. AFs damage mitochondrial respiratory function through modifying mtDNA and mitochondrial respiratory complexes I-IV, which generates ROS and extensive oxidative stress. Comparatively speaking, AFs destroy Keap1/Nrf2/ARE antioxidant pathway to amplify oxidative stress of hepatocytes further. On the other hand, AFs damage the structure of mitochondria through regulating Bax and opening mPTP. AFs induce the translocation of Bax to mitochondria through regulating Bcl-2 family proteins and p53 to generate lipid pores within OMM thereby damaging the structure of mitochondria. And damaged mitochondria initiate mitophagy by COX-2. The AFs-induced opening of mPTP together with the translocation of Bax make mitochondria release contents such as cyt c. The activation of death receptor (extrinsic pathway) and released cyt c from damaged mitochondria (intrinsic pathway) induce apoptosis through activating caspase cascade reaction. Meanwhile, AFs alter ER localization of COX-2 by phosphatase PP2A-B55δ to promote NLRP3 inflammasome activation, furthermore, NLRP3 inflammasome activates caspase-1 (induce pyroptosis of hepatocytes) and converts pro-IL-1β into mature form. AFs also activate NF-κB signaling pathway to secrete cytokines such as TNF and IL via TLR. Released DAMPs and cytokines from hepatocytes interact with KCs (liver innate immunocytes) that also have toxic effects for hepatocytes. The interaction between hepatocytes and KCs forms a vice feedback loop to exasperate local hepatic inflammatory microenvironment.

Scientific studies have made increasing progress in understanding the mechanisms of AFs-induced hepatotoxicity. Due to the multifactorial and complicated toxicology involved, a better understanding of the mechanisms of AFs-induced hepatotoxicity is a challenge to solve the problem of AFs contamination and provide a basis for clinical purpose. Under exposure of AFs in almost all of edible foods and herbal medicines chronically or acutely, AFs induce hepatotoxicity after metabolic bioactivation in liver cells through destroying mitochondria, inhibiting cellular protective processes, further cell death and local hepatic inflammatory response (Figure 3).

Considering the widespread contamination of AFs in foods and herbal medicines, which have strong toxicity for liver, many problems are waiting for us to complement the pathway network of AFs-related hepatotoxicity. Firstly, more precise evidence is needed to elucidate how the mitochondrial genome influences mitochondial OXPHOS function after exposure to AFs. Further studies are required to clarify how AFs exactly influence lipid metabolism because many studies reported that AFs induce lipid droplets, lipid accumulation and steatosis (Shi et al., 2012a; El-Nekeety et al., 2014; Shi et al., 2015; Rotimi et al., 2017; Muhammad et al., 2018a; Wang et al., 2018). Secondly, the role of AFs in the autophagy of hepatocytes must be substantiated under different conditions, such as different doses and types of AFs, and different experimental models. In contrast, evidence of necrosis-related cell death is not clear in AFs-induced hepatotoxicity. Lastly, how the effects of AFs on the immune system impact the AFs-induced inflammatory response need to be clarified (Abbès et al., 2010; Abbès et al., 2016). For example, AFs inhibit phagocytosis, microbicidal activity and intrinsic antiviral activity of KCs, and how these effects impact AFs-induced hepatotoxicity is not known (Mohapatra and Roberts, 1985; Cusumano et al., 1995)? What are specific evidences about AFs-induced fibrosis in hepatic tissue (Yener et al., 2009; Chen et al., 2019b; Owumi et al., 2020)?

## Author Contributions

ZH wrote the whole review including all figures and tables; YL, CL and WL directed writing; YC, GL, CL, YS and WL, specially, RL and ZC provided valuable suggestions about writing and drawing.

## Funding

This work was supported by Beijing Natural Science Foundation (7202111) and National Science and Technology Major Project (2018ZX10101001–005–003) and it also supported by the Fundamental Research Funds for the Central public welfare research institutes (Z0653/Z0656). All special thanks for the long-term subsidy mechanism from the Ministry of Finance and the Ministry of Education of PRC for BUCM.

## Conflict of Interest

The authors declare that the research was conducted in the absence of any commercial or financial relationships that could be construed as a potential conflict of interest.

## References

[B1] Abbès S. Ben Salah-Abbès J. Abdel-Wahhab M. Ouslati R. (2010). Immunotoxicological and biochemical effects of aflatoxins in rats prevented by Tunisian montmorillonite with reference to HSCAS. Immunopharmacol. Immunotoxicol. 32, 514–522. 10.3109/08923970903440176 20088648

[B2] Abbès S. Ben Salah-Abbès J. Jebali R. Younes R. Oueslati R. (2016). Interaction of aflatoxin B1 and fumonisin B1 in mice causes immunotoxicity and oxidative stress: possible protective role using lactic acid bacteria. J. Immunot. 13, 46–54. 10.3109/1547691x.2014.997905 25585958

[B3] Abdel-Aziem S. Hassan A. El-Denshary E. Hamzawy M. Mannaa F. Abdel-Wahhab M. (2014). Ameliorative effects of thyme and calendula extracts alone or in combination against aflatoxins-induced oxidative stress and genotoxicity in rat liver. Cytotechnol. 66, 457–470. 10.1007/s10616-013-9598-7 PMC3973790 24096837

[B4] Adeleye A. Ajiboye T. Iliasu G. Abdussalam F. Balogun A. Ojewuyi O. (2014). Phenolic extract of Dialium guineense pulp enhances reactive oxygen species detoxification in aflatoxin B₁ hepatocarcinogenesis. J. Med. Food 17, 875–885. 10.1089/jmf.2013.0157 24892362

[B5] Afonina I. Zhong Z. Karin M. Beyaert R. (2017). Limiting inflammation-the negative regulation of NF-κB and the NLRP3 inflammasome. Nat. Immunol. 18, 861–869. 10.1038/ni.3772 28722711

[B6] Afshar P. Shokrzadeh M. Raeisi S. Ghorbani-HasanSaraei A. Nasiraii L. (2020). Aflatoxins biodetoxification strategies based on probiotic bacteria. Toxicon 178, 50–58. 10.1016/j.toxicon.2020.02.007 32250747

[B7] Ajiboye T. Yakubu M. Oladiji A. (2016). Lophirones B and C prevent aflatoxin B1-induced oxidative stress and DNA fragmentation in rat hepatocytes. Pharmaceut. Biol. 54, 1962–1970. 10.3109/13880209.2015.1137603 26841338

[B8] Akçam M. Artan R. Yilmaz A. Ozdem S. Gelen T. Nazıroğlu M. (2013). Caffeic acid phenethyl ester modulates aflatoxin B1-induced hepatotoxicity in rats. Cell Biochem. Funct. 31, 692–697. 10.1002/cbf.2957 23400894

[B9] Alehashemi S. Goldbach-Mansky R. (2020). Human autoinflammatory diseases mediated by NLRP3-, pyrin-, NLRP1-, and NLRC4-inflammasome dysregulation updates on diagnosis, treatment, and the respective roles of IL-1 and IL-18. Front. Immunol. 11, 1840 10.3389/fimmu.2020.01840 32983099 PMC7477077

[B10] Ali Rajput S. Sun L. Zhang N. Mohamed Khalil M. Gao X. Ling Z. (2017). Ameliorative effects of grape seed proanthocyanidin extract on growth performance, immune function, antioxidant capacity, biochemical constituents, liver histopathology and aflatoxin residues in broilers exposed to aflatoxin B₁. Toxins 9, 371 10.3390/toxins9110371 PMC5705986 29140290

[B11] Alm-Eldeen A. Basyony M. Elfiky N. Ghalwash M. (2017). Effect of the Egyptian propolis on the hepatic antioxidant defense and pro-apoptotic p53 and anti-apoptotic bcl2 expressions in aflatoxin B1 treated male mice. Biomed. Pharmacother. 87, 247–255. 10.1016/j.biopha.2016.12.084 28063405

[B12] Alm-Eldeen A. Mona M. Shati A. El-Mekkawy H. (2015). Synergistic effect of black tea and curcumin in improving the hepatotoxicity induced by aflatoxin B1 in rats. Toxicol. Ind. Health 31, 1269–1280. 10.1177/0748233713491807 23796760

[B13] An Y. Shi X. Tang X. Wang Y. Shen F. Zhang Q. (2017). Aflatoxin B1 induces reactive oxygen species-mediated autophagy and extracellular trap formation in macrophages. F. Cellular Infect. Microbiol. 7, 53 10.3389/fcimb.2017.00053 PMC5322174 28280716

[B116] A V V. K. R. R. Kurrey N. K. K A A. A. G V. (2017). Protective effects of phenolics rich extract of ginger against Aflatoxin B-induced oxidative stress and hepatotoxicity. Biomed. Pharmacother. 91, 415–424. 10.1016/j.biopha.2017.04.107 28475920

[B14] Ayed-Boussema I. Pascussi J. Maurel P. Bacha H. Hassen W. (2012). Effect of aflatoxin B1 on nuclear receptors PXR, CAR, and AhR and their target cytochromes P450 mRNA expression in primary cultures of human hepatocytes. Int. J. Toxicol. 31, 86–93. 10.1177/1091581811422453 21994236

[B15] Baan R. Grosse Y. Straif K. Secretan B. El Ghissassi F. Bouvard V. (2009). A review of human carcinogens--Part F: chemical agents and related occupations. Lancet Oncol. 10, 1143–1144. 10.1016/s1470-2045(09)70358-4 19998521

[B16] Baines C. Gutiérrez-Aguilar M. (2018). The still uncertain identity of the channel-forming unit(s) of the mitochondrial permeability transition pore. Cell Calcium 73, 121–130. 10.1016/j.ceca.2018.05.003 29793100 PMC5993635

[B17] Begriche K. Massart J. Robin M. Borgne-Sanchez A. Fromenty B. (2011). Drug-induced toxicity on mitochondria and lipid metabolism: mechanistic diversity and deleterious consequences for the liver. J. Hepatol. 54, 773–794. 10.1016/j.jhep.2010.11.006 21145849

[B19] Bock F. Tait S. (2020). Mitochondria as multifaceted regulators of cell death. Nat. Rev. Mol. Cell Biol. 21, 85–100. 10.1038/s41580-019-0173-8 31636403

[B20] Brenner D. Mak T. (2009). Mitochondrial cell death effectors. Curr. Opin. Cell Biol. 21, 871–877. 10.1016/j.ceb.2009.09.004 19822411

[B21] Briston T. Selwood D. Szabadkai G. Duchen M. (2019). Mitochondrial permeability transition: a molecular lesion with multiple drug targets. Trends Pharmacol. Sci. 40, 50–70. 10.1016/j.tips.2018.11.004 30527591

[B22] Bryan H. Olayanju A. Goldring C. Park B. (2013). The Nrf2 cell defence pathway: Keap1-dependent and -independent mechanisms of regulation. Biochem. Pharmacol. 85, 705–717. 10.1016/j.bcp.2012.11.016 23219527

[B23] Chalah A. Khosravi-Far R. (2008). The mitochondrial death pathway. Adv. Exp. Med. Biol. 615, 25–45. 10.1007/978-1-4020-6554-5_3 18437890

[B24] Chen B. Li D. Li M. Li S. Peng K. Shi X. (2016). Induction of mitochondria-mediated apoptosis and PI3K/Akt/mTOR-mediated autophagy by aflatoxin B2 in hepatocytes of broilers. Oncotarget 7, 84989–84998. 10.18632/oncotarget.13356 27863407 PMC5356714

[B25] Chen Y. Li R. Chang Q. Dong Z. Yang H. Xu C., (2019a). Lactobacillus bulgaricus or suppresses NF-κB signaling pathway and protects against AFB₁-induced hepatitis: a novel potential preventive strategy for aflatoxicosis? Toxins 11, 17 10.3390/toxins11010017 PMC6356522 30621122

[B26] Chen Y. Lin Y. Han P. Jiang S. Che L. He C. (2019b). HBx combined with AFB1 triggers hepatic steatosis via COX-2-mediated necrosome formation and mitochondrial dynamics disorder. J. Cell Mol. Med. 23, 5920–5933. 10.1111/jcmm.14388 31282064 PMC6714226

[B27] Cohen A. Hnasko R. Schubert W. Lisanti M. (2004). Role of caveolae and caveolins in health and disease. Physiol. Rev. 84, 1341–1379. 10.1152/physrev.00046.2003 15383654

[B28] Cotty P. Jaime-Garcia R. (2007). Influences of climate on aflatoxin producing fungi and aflatoxin contamination. Int. J. Food Microbiol. 119, 109–115. 10.1016/j.ijfoodmicro.2007.07.060 17881074

[B29] Cusumano V. Costa G. Trifiletti R. Merendino R. Mancuso G. (1995). Functional impairment of rat Kupffer cells induced by aflatoxin B1 and its metabolites. FEMS Immun.Med. Microbiol. 10, 151–155. 10.1111/j.1574-695X.1995.tb00025.x 7719284

[B30] D'Ovidio K. Trucksess M. Weaver C. Horn E. McIntosh M. Bean G. (2006). Aflatoxins in ginseng roots. Food Addit. Contam. 23, 174–180. 10.1080/02652030500442524 16449060

[B31] Deng J. Zhao L. Zhang N. Karrow N. Krumm C. Qi D. (2018). Aflatoxin B metabolism: regulation by phase I and II metabolizing enzymes and chemoprotective agents. Mutat. Res. 778, 79–89. 10.1016/j.mrrev.2018.10.002 30454686

[B32] Deng Z. Zhao J. Huang F. Sun G. Gao W. Lu L. (2020). Protective effect of procyanidin B2 on acute liver injury induced by aflatoxin B in rats. Biomed. Environ. Sci. 33, 238–247. 10.3967/bes2020.033 32438961

[B33] Dohnal V. Wu Q. Kuča K. (2014). Metabolism of aflatoxins: key enzymes and interindividual as well as interspecies differences. Arch. Toxicol. 88, 1635–1644. 10.1007/s00204-014-1312-9 25027283

[B34] Dowling J. O'Neill L. (2012). Biochemical regulation of the inflammasome. Crit. Rev. Biochem. Mol. Biol. 47, 424–443. 10.3109/10409238.2012.694844 22681257

[B35] Eaton D. Gallagher E. (1994). Mechanisms of aflatoxin carcinogenesis. Annu. Rev. Pharmacol. Toxicol. 34, 135–172. 10.1146/annurev.pa.34.040194.001031 8042848

[B36] Eftekhari A. Ahmadian E. Panahi-Azar V. Hosseini H. Tabibiazar M. Maleki Dizaj S. (2018). Hepatoprotective and free radical scavenging actions of quercetin nanoparticles on aflatoxin B1-induced liver damage: *in vitro*/*in vivo* studies. Artif. cells Nanomed. Biotechnol. 46, 411–420. 10.1080/21691401.2017.1315427 28423950

[B37] El-Nekeety A. Abdel-Azeim S. Hassan A. Hassan N. Aly S. Abdel-Wahhab M. (2014). Quercetin inhibits the cytotoxicity and oxidative stress in liver of rats fed aflatoxin-contaminated diet. Toxicol. Reports 1, 319–329. 10.1016/j.toxrep.2014.05.014 PMC5598466 28962248

[B38] Erdélyi M. Balogh K. Pelyhe C. Kövesi B. Nakade M. Zándoki E. (2018). Changes in the regulation and activity of glutathione redox system, and lipid peroxidation processes in short-term aflatoxin B1 exposure in liver of laying hens. J. Anim. Physiol. Anim. Nutr. 102, 947–952. 10.1111/jpn.12896 29604131

[B39] Essa S. El-Saied E. El-Tawil O. Mahmoud M. Abd El-Rahman S. (2017). Modulating effect of MgO-SiO nanoparticles on immunological and histopathological alterations induced by aflatoxicosis in rats. Toxicon 140, 94–104. 10.1016/j.toxicon.2017.10.018 29079028

[B40] Fang Y. Feng Y. Wu T. Srinivas S. Yang W. Fan J. (2013). Aflatoxin B1 negatively regulates Wnt/β-catenin signaling pathway through activating miR-33a. PloS One 8, e73004 10.1371/journal.pone.0073004 24015284 PMC3754916

[B41] Gan F. Yang Y. Chen Y. Che C. Pan C. Huang K. (2018). Bush sophora root polysaccharide could help prevent aflatoxin B1-induced hepatotoxicity in the primary chicken hepatocytes. Toxicon 150, 180–187. 10.1016/j.toxicon.2018.05.019 29857086

[B42] Gaschler M. Stockwell B. (2017). Lipid peroxidation in cell death. Biochem. Biophys. Res. Commun. 482, 419–425. 10.1016/j.bbrc.2016.10.086 28212725 PMC5319403

[B43] Gesing A. Karbownik-Lewinska M. (2008). Protective effects of melatonin and N-acetylserotonin on aflatoxin B1-induced lipid peroxidation in rats. Cell Biochem. Funct. 26, 314–319. 10.1002/cbf.1438 17868196

[B44] Green D. Llambi F., 2015 Cell death signaling. Cold Spring Harb. Perspect. Biol. 7, a006080 10.1101/cshperspect.a006080 26626938 PMC4665079

[B45] Guillot A. Tacke F. (2019). Liver macrophages: old dogmas and new insights. Hepatol. Commun. 3, 730–743. 10.1002/hep4.1356 31168508 PMC6545867

[B46] Hamid A. Tesfamariam I. Zhang Y. Zhang Z. (2013). Aflatoxin B1-induced hepatocellular carcinoma in developing countries: geographical distribution, mechanism of action and prevention. Oncol. Lett. 5, 1087–1092. 10.3892/ol.2013.1169 23599745 PMC3629261

[B47] Hu S. Dou X. Zhang L. Xie Y. Yang S. Yang M. (2018). Rapid detection of aflatoxin B in medicinal materials of radix and rhizome by gold immunochromatographic assay. Toxicon 150, 144–150. 10.1016/j.toxicon.2018.05.015 29800608

[B48] Huang L. Zhao Z. Duan C. Wang C. Zhao Y. Yang G. (2019). Lactobacillus plantarum C88 protects against aflatoxin B-induced liver injury in mice via inhibition of NF-κB-mediated inflammatory responses and excessive apoptosis. BMC Microbiol. 19, 170 10.1186/s12866-019-1525-4 31357935 PMC6664579

[B49] Jaeschke H. McGill M. Ramachandran A. (2012). Oxidant stress, mitochondria, and cell death mechanisms in drug-induced liver injury: lessons learned from acetaminophen hepatotoxicity. Drug Metabol. Reviews 44, 88–106. 10.3109/03602532.2011.602688 PMC5319847 22229890

[B50] Ji Y. Nyamagoud S. SreeHarsha N. Mishra A. Gubbiyappa S. Singh Y. (2020). Sitagliptin protects liver against aflatoxin B1-induced hepatotoxicity through upregulating Nrf2/ARE/HO-1 pathway. Biofactors 46, 76–82. 10.1002/biof.1573 31600004

[B51] Jowsey I. Jiang Q. Itoh K. Yamamoto M. Hayes J. (2003). Expression of the aflatoxin B1-8,9-epoxide-metabolizing murine glutathione S-transferase A3 subunit is regulated by the Nrf2 transcription factor through an antioxidant response element. Mol. Pharmacol. 64, 1018–1028. 10.1124/mol.64.5.1018 14573750

[B52] Karaman M. Ozen H. Tuzcu M. Ciğremiş Y. Onder F. Ozcan K. (2010). Pathological, biochemical and haematological investigations on the protective effect of alpha-lipoic acid in experimental aflatoxin toxicosis in chicks. Br. Poultry Sci. 51, 132–141. 10.1080/00071660903401839 20390578

[B53] Kensler K. Slocum S. Chartoumpekis D. Dolan P. Johnson N. Ilic Z. (2014). Genetic or pharmacologic activation of Nrf2 signaling fails to protect against aflatoxin genotoxicity in hypersensitive GSTA3 knockout mice. Toxicol. Sci. 139, 293–300. 10.1093/toxsci/kfu056 24675090 PMC4064015

[B54] Kensler T. Roebuck B. Wogan G. Groopman J. (2011). Aflatoxin: a 50-year odyssey of mechanistic and translational toxicology. Toxicol. Sci. 120, S28–S48. 10.1093/toxsci/kfq283 20881231 PMC3043084

[B55] Kong W. Li J. Qiu F. Wei J. Xiao X. Zheng Y. (2013). Development of a sensitive and reliable high performance liquid chromatography method with fluorescence detection for high-throughput analysis of multi-class mycotoxins in Coix seed. Anal. Chim. Acta 799, 68–76. 10.1016/j.aca.2013.08.042 24091376

[B56] Koohi Mohammad K. Ghazi-Khansari M. Hayati F. Staji H. Keywanloo M. Shahroozian E. (2017). The role of TNF-α in aflatoxin B-1 induced hepatic toxicity in isolated perfused rat liver model. Acta Med. Iran. 55, 416–421. 28918610

[B57] Kövesi B. Pelyhe C. Zándoki E. Mézes M. Balogh K. (2018). Changes of lipid peroxidation and glutathione redox system, and expression of glutathione peroxidase regulatory genes as effect of short-term aflatoxin B exposure in common carp. Toxicon 144, 103–108. 10.1016/j.toxicon.2018.02.003 29453995

[B58] Krenkel O. Tacke F. (2017). Liver macrophages in tissue homeostasis and disease. Nat. Rev. Immunol. 17, 306–321. 10.1038/nri.2017.11 28317925

[B59] Kubes P. Mehal W. (2012). Sterile inflammation in the liver. Gastroenterol. 143, 1158–1172. 10.1053/j.gastro.2012.09.008 22982943

[B60] Kumagai T. Kawamoto Y. Nakamura Y. Hatayama I. Satoh K. Osawa T. (2000). 4-hydroxy-2-nonenal, the end product of lipid peroxidation, is a specific inducer of cyclooxygenase-2 gene expression. Biochem. Biophys. Res. Commun. 273, 437–441. 10.1006/bbrc.2000.2967 10873624

[B61] Kumar P. Mahato D. Kamle M. Mohanta T. Kang S. (2016). Aflatoxins: a global concern for food safety, human health and their management. Front. Microbiol. 7, 2170 10.3389/fmicb.2016.02170 28144235 PMC5240007

[B62] Li S. Muhammad I. Yu H. Sun X. Zhang X. (2019). Detection of Aflatoxin adducts as potential markers and the role of curcumin in alleviating AFB1-induced liver damage in chickens. Ecotoxicol. Environ. Saf. 176, 137–145. 10.1016/j.ecoenv.2019.03.089 30925330

[B63] Li Y. Ma Q. Zhao L. Guo Y. Duan G. Zhang J. (2014). Protective efficacy of alpha-lipoic acid against AflatoxinB1-induced oxidative damage in the liver. Asian-Australas. J. Anim. Sci. 27, 907–915. 10.5713/ajas.2013.13588 25050030 PMC4093170

[B64] Liao S. Shi D. Clemons-Chevis C. Guo S. Su R. Qiang P. (2014). Protective role of selenium on aflatoxin b1-induced hepatic dysfunction and apoptosis of liver in ducklings. Biol. Trace Elem. Res. 162, 296–301. 10.1007/s12011-014-0131-4 25274191

[B65] Lin L. Baehrecke E. (2015). Autophagy, cell death, and cancer. Mol. Cellular Oncol. 2, e985913 10.4161/23723556.2014.985913 PMC4905302 27308466

[B66] Lin Y. Li L. Makarova A. Burgers P. Stone M. Lloyd R. (2014). Error-prone replication bypass of the primary aflatoxin B1 DNA adduct, AFB1-N7-Gua. J. Biol. Chem. 289, 18497–18506. 10.1074/jbc.M114.561563 24838242 PMC4140297

[B67] Lin Y. Owen N. Minko I. Lange S. Tomida J. Li L. (2016). DNA polymerase ζ limits chromosomal damage and promotes cell survival following aflatoxin exposure. Proc. Natl. Acad. Sci. U.S.A. 113, 13774–13779. 10.1073/pnas.1609024113 27849610 PMC5137696

[B68] Liu C. Yu H. Zhang Y. Li D. Xing X. Chen L. (2015). Upregulation of miR-34a-5p antagonizes AFB1-induced genotoxicity in F344 rat liver. Toxicon 106, 46–56. 10.1016/j.toxicon.2015.09.016 26385312

[B69] Liu J. Sun L. Zhang N. Zhang J. Guo J. Li C. (2016). Effects of nutrients in substrates of different grains on aflatoxin B1 production by Aspergillus flavus. BioMed. Res. Int., 7232858 10.1155/2016/7232858 27294129 PMC4886045

[B70] Liu Y. Chang C. Marsh G. Wu F. (2012). Population attributable risk of aflatoxin-related liver cancer: systematic review and meta-analysis. Eur. J. Cancer 48, 2125–2136. 10.1016/j.ejca.2012.02.009 22405700 PMC3374897

[B71] Liu Y. Wang W. (2016). Aflatoxin B1 impairs mitochondrial functions, activates ROS generation, induces apoptosis and involves Nrf2 signal pathway in primary broiler hepatocytes. Ani. Sci. J. 87, 1490–1500. 10.1111/asj.12550 26997555

[B72] Livingstone M. Johnson N. Roebuck B. Kensler T. Groopman J. (2017). Profound changes in miRNA expression during cancer initiation by aflatoxin B and their abrogation by the chemopreventive triterpenoid CDDO-Im. Mol. Carcinog. 56, 2382–2390. 10.1002/mc.22635 28218475 PMC5563488

[B73] Ma Q. Li Y. Fan Y. Zhao L. Wei H. Ji C. (2015). Molecular mechanisms of lipoic acid protection against aflatoxin B₁-Induced liver oxidative damage and inflammatory responses in broilers. Toxins 7, 5435–5447. 10.3390/toxins7124879 26694462 PMC4690129

[B74] Mahuku G. Nzioki H. Mutegi C. Kanampiu F. Narrod C. Makumbi D. (2019). Pre-harvest management is a critical practice for minimizing aflatoxin contamination of maize. Food Control 96, 219–226. 10.1016/j.foodcont.2018.08.032 30713368 PMC6251936

[B75] Malhi H. Gores G. Lemasters J. (2006). Apoptosis and necrosis in the liver: a tale of two deaths? Hepatology 43, S31–S44. 10.1002/hep.21062 16447272

[B76] Mao J. He B. Zhang L. Li P. Zhang Q. Ding X. (2016). A structure identification and toxicity assessment of the degradation products of aflatoxin B₁ in peanut oil under UV irradiation. Toxins 8, 332 10.3390/toxins8110332 PMC5127128 27845743

[B77] Marrone A. Tryndyak V. Beland F. Pogribny I. (2016). MicroRNA responses to the genotoxic carcinogens aflatoxin B1 and benzo[a]pyrene in human HepaRG cells. Toxicol. Sci. 149, 496–502. 10.1093/toxsci/kfv253 26609139

[B78] Mary V. Theumer M. Arias S. Rubinstein H. (2012). Reactive oxygen species sources and biomolecular oxidative damage induced by aflatoxin B1 and fumonisin B1 in rat spleen mononuclear cells. Toxicol. 302, 299–307. 10.1016/j.tox.2012.08.012 22981896

[B79] Mary V. Valdehita A. Navas J. Rubinstein H. Fernández-Cruz M. (2015). Effects of aflatoxin B₁, fumonisin B₁ and their mixture on the aryl hydrocarbon receptor and cytochrome P450 1A induction. Food Chem. Toxicol. 75, 104–111. 10.1016/j.fct.2014.10.030 25449202

[B80] Maurya B. Trigun S. (2016). Fisetin modulates antioxidant enzymes and inflammatory factors to inhibit aflatoxin-B1 induced hepatocellular carcinoma in rats. Oxid. Med. Cell. Longev. 2016, 1972793 10.1155/2016/1972793 26682000 PMC4670673

[B81] Mohapatra N. Roberts J. (1985). *In vitro* effect of aflatoxin B1 on rat liver macrophages (Kuffer cells). Toxicol. Lett. 29, 177–181. 10.1016/0378-4274(85)90039-6 2418540

[B82] Mughal M. Xi P. Yi Z. Jing F. (2017). Aflatoxin B1 invokes apoptosis via death receptor pathway in hepatocytes. Oncotarget 8, 8239–8249. 10.18632/oncotarget.14158 28030812 PMC5352397

[B83] Muhammad I. Wang H. Sun X. Wang X. Han M. Lu Z. (2018a). Dual role of dietary curcumin through attenuating AFB-induced oxidative stress and liver injury via modulating liver phase-I and phase-II enzymes involved in AFB bioactivation and detoxification. Front. Pharmacol. 9, 554 10.3389/fphar.2018.00554 29887802 PMC5981209

[B84] Muhammad I. Wang X. Li S. Li R. Zhang X. (2018b). Curcumin confers hepatoprotection against AFB-induced toxicity via activating autophagy and ameliorating inflammation involving Nrf2/HO-1 signaling pathway. Mol. Biol. Rep. 45, 1775–1785. 10.1007/s11033-018-4323-4 30143976

[B85] Nian Y. Wang H. Ying G. Yang M. Wang Z. Kong W. (2018). Transfer rates of aflatoxins from herbal medicines to decoctions determined by an optimized high-performance liquid chromatography with fluorescence detection method. J. Pharm. Pharmacol. 70, 278–288. 10.1111/jphp.12856 29193086

[B86] Oskoueian E. Abdullah N. Zulkifli I. Ebrahimi M. Karimi E. Goh Y. (2015). Cytoprotective effect of palm kernel cake phenolics against aflatoxin B1-induced cell damage and its underlying mechanism of action. BMC Complement. Alter. Med. 15, 392 10.1186/s12906-015-0921-z PMC4628249 26518905

[B87] Ou C. Zheng H. Su J. Cao J. Li G. Li L. (2014). Effect of Ginkgo biloba extract on the expressions of Cox-2 and GST-Pi in rats with hepatocellular carcinoma risk. Afr. Health Sci. 14, 37–48. 10.4314/ahs.v14i1.7 26060456 PMC4449074

[B88] Owumi S. Najophe E. Farombi E. Oyelere A. (2020). Gallic acid protects against Aflatoxin B -induced oxidative and inflammatory stress damage in rats kidneys and liver. J. Food Biochem. 44, e13316 10.1111/jfbc.13316 32496616

[B89] Ozen H. Karaman M. Ciğremiş Y. Tuzcu M. Ozcan K. Erdağ D. (2009). Effectiveness of melatonin on aflatoxicosis in chicks. Res. Vet. Sci. 86, 485–489. 10.1016/j.rvsc.2008.09.011 19036390

[B90] Pandey M. Kumar R. Pandey A. Soni P. Gangurde S. Sudini H. (2019). Mitigating aflatoxin contamination in groundnut through A combination of genetic resistance and post-harvest management practices. Toxins 11, 315 10.3390/toxins11060315 PMC6628460 31163657

[B91] Pessayre D. Fromenty B. Berson A. Robin M. Lettéron P. Moreau R. (2012). Central role of mitochondria in drug-induced liver injury. Drug Metabol. Reviews 44, 34–87. 10.3109/03602532.2011.604086 21892896

[B92] Prandini A. Tansini G. Sigolo S. Filippi L. Laporta M. Piva G. (2009). On the occurrence of aflatoxin M1 in milk and dairy products. Food Chem. Toxicol. 47, 984–991. 10.1016/j.fct.2007.10.005 18037552

[B93] Qin H. Li H. Zhou X. Peng C. Tan H. Wang M. (2016). Effect of superoxide and inflammatory factor on aflatoxin B1 triggered hepatocellular carcinoma. Am. J. Tourism Res. 8, 4003–4008. PMC5040699 27725881

[B94] Rajput S. Sun L. Zhang N. Khalil M. Ling Z. Chong L. (2019). Grape seed proanthocyanidin extract alleviates AflatoxinB₁-induced immunotoxicity and oxidative stress via modulation of NF-κB and Nrf2 signaling pathways in broilers. Toxins 11, 23 10.3390/toxins11010023 PMC6356337 30621062

[B95] Raysyan A. Eremin S. Beloglazova N. De Saeger S. Gravel I. (2020). Immunochemical approaches for detection of aflatoxin B1 in herbal medicines. Phytochem. Anal. 31, 662–669. 10.1002/pca.2931 32150783

[B96] Ren X. Han P. Meng Y. (2020). Aflatoxin B1-induced COX-2 expression promotes mitophagy and contributes to lipid accumulation in hepatocytes in vitro and in vivo. Int. J. Toxicol. 39, 1091581820939081 10.1177/1091581820939081 32687719

[B97] Rotimi O. Rotimi S. Duru C. Ebebeinwe O. Abiodun A. Oyeniyi B. (2017). Acute aflatoxin B1 - induced hepatotoxicity alters gene expression and disrupts lipid and lipoprotein metabolism in rats. Toxicol. Reports 4, 408–414. 10.1016/j.toxrep.2017.07.006 PMC5615163 28959666

[B98] Rushing B. Selim M. (2019). Aflatoxin B1: a review on metabolism, toxicity, occurrence in food, occupational exposure, and detoxification methods. Food Chem. Toxicol. 124, 81–100. 10.1016/j.fct.2018.11.047 30468841

[B99] Sanjay S. Girish C. (2017). Role of miRNA and its potential as a novel diagnostic biomarker in drug-induced liver injury. Eur. J. Clin. Pharmacol. 73, 399–407. 10.1007/s00228-016-2183-1 28028586

[B100] Sheehan D. Meade G. Foley V. Dowd C. (2001). Structure, function and evolution of glutathione transferases: implications for classification of non-mammalian members of an ancient enzyme superfamily. Biochem. J. 360, 1–16. 10.1042/0264-6021:3600001 11695986 PMC1222196

[B101] Shi D. Guo S. Liao S. Su R. Guo M. Liu N. (2012a). Protection of selenium on hepatic mitochondrial respiratory control ratio and respiratory chain complex activities in ducklings intoxicated with aflatoxin B₁. Biol. Trace Elem. Res. 145, 312–317. 10.1007/s12011-011-9195-6 21909799

[B102] Shi D. Guo S. Liao S. Su R. Pan J. Lin Y. (2012b). Influence of selenium on hepatic mitochondrial antioxidant capacity in ducklings intoxicated with aflatoxin B₁. Biol. Trace Elem. Res. 145, 325–329. 10.1007/s12011-011-9201-z 21935652

[B103] Shi D. Liao S. Guo S. Li H. Yang M. Tang Z. (2015). Protective effects of selenium on aflatoxin B1-induced mitochondrial permeability transition, DNA damage, and histological alterations in duckling liver. Biol. Trace Elem. Res. 163, 162–168. 10.1007/s12011-014-0189-z 25431300

[B104] Singh K. Maurya B. Trigun S. (2015). Activation of oxidative stress and inflammatory factors could account for histopathological progression of aflatoxin-B1 induced hepatocarcinogenesis in rat. Mol. Cell. Biochem. 401, 185–196. 10.1007/s11010-014-2306-x 25543524

[B105] Sun L. Lei M. Zhang N. Gao X. Li C. Krumm C. (2015a). Individual and combined cytotoxic effects of aflatoxin B1, zearalenone, deoxynivalenol and fumonisin B1 on BRL 3A rat liver cells. Toxicon 95, 6–12. 10.1016/j.toxicon.2014.12.010 25549941

[B106] Sun L. Zhang N. Zhu M. Zhao L. Zhou J. Qi D. (2015b). Prevention of aflatoxin B1 hepatoxicity by dietary selenium is associated with inhibition of cytochrome P450 isozymes and up-regulation of 6 selenoprotein genes in chick liver. J. Nutr. 146, 655–661. 10.3945/jn.115.224626 26962192

[B107] Tacke F. Zimmermann H. (2014). Macrophage heterogeneity in liver injury and fibrosis. J. Hepatol. 60, 1090–1096. 10.1016/j.jhep.2013.12.025 24412603

[B108] Taguchi K. Takaku M. Egner P. Morita M. Kaneko T. Mashimo T. (2016). Generation of a new model rat: Nrf2 knockout rats are sensitive to aflatoxin B1 toxicity. Toxicol. Sci. 152, 40–52 10.1093/toxsci/kfw065 27071940 PMC4922541

[B109] Taranu I. Marin D. Palade M. Pistol G. Chedea V. Gras M. (2019). Assessment of the efficacy of a grape seed waste in counteracting the changes induced by aflatoxin B1 contaminated diet on performance, plasma, liver and intestinal tissues of pigs after weaning. Toxicon 162, 24–31. 10.1016/j.toxicon.2019.02.020 30849456

[B110] Tu W. Wang H. Li S. Liu Q. Sha H. (2019). The anti-inflammatory and anti-oxidant mechanisms of the keap1/nrf2/ARE signaling pathway in chronic diseases. Aging Disease 10, 637–651. 10.14336/ad.2018.0513 31165007 PMC6538222

[B111] Turner N. Subrahmanyam S. Piletsky S. (2009). Analytical methods for determination of mycotoxins: a review. Anal. Chim. Acta 632, 168–180. 10.1016/j.aca.2008.11.010 19110091

[B112] Valavanidis A. Vlachogianni T. Fiotakis C. (2009). 8-hydroxy-2' -deoxyguanosine (8-OHdG): a critical biomarker of oxidative stress and carcinogenesis. J. Environ. Sci. Health C Environ. Carcinog. Ecotoxicol. Rev. 27, 120–139. 10.1080/10590500902885684 19412858

[B113] Ventura M. Gómez A. Anaya I. Díaz J. Broto F. Agut M. (2004). Determination of aflatoxins B1, G1, B2 and G2 in medicinal herbs by liquid chromatography-tandem mass spectrometry. J. Chromatogr. A 1048, 25–29. 10.1016/s0021-9673(04)01188-4 15453415

[B114] Verheecke C. Liboz T. Mathieu F. (2016). Microbial degradation of aflatoxin B1: current status and future advances. Int. J. Food Microbiol. 237, 1–9. 10.1016/j.ijfoodmicro.2016.07.028 27541976

[B115] Verma R. Chakraborty B. Patel C. Mathuria N. (2008). Curcumin ameliorates aflatoxin-induced changes in SDH and ATPase activities in liver and kidney of mice. Acta Pol. Pharm. 65, 415–419. 19051581

[B117] Vogel C. Van Winkle L. Esser C. Haarmann-Stemmann T. (2020). The aryl hydrocarbon receptor as a target of environmental stressors–implications for pollution mediated stress and inflammatory responses. Redox Biol. 34, 101530 10.1016/j.redox.2020.101530 32354640 PMC7327980

[B118] Wang X. Li W. Wang X. Han M. Muhammad I. Zhang X. (2019). Water-soluble substances of wheat: a potential preventer of aflatoxin B1-induced liver damage in broilers. Poultry Sci. 98, 136–149. 10.3382/ps/pey358 30107611

[B119] Wang X. Muhammad I. Sun X. Han M. Hamid S. Zhang X. (2018). Protective role of curcumin in ameliorating AFB-induced apoptosis via mitochondrial pathway in liver cells. Mol. Biol. Rep. 45, 881–891. 10.1007/s11033-018-4234-4 29974318

[B120] Wen J. Kong W. Wang J. Yang M. (2013). Simultaneous determination of four aflatoxins and ochratoxin A in ginger and related products by HPLC with fluorescence detection after immunoaffinity column clean-up and postcolumn photochemical derivatization. J. Separ. Sci. 36, 3709–3716. 10.1002/jssc.201300885 24115567

[B121] Wogan G. Kensler T. Groopman J. (2012). Present and future directions of translational research on aflatoxin and hepatocellular carcinoma. A review. Food Addit. Contam. Part A Chem. Anal. Control Exp. Risk Assess. 29, 249–257. 10.1080/19440049.2011.563370 PMC4659374 21623489

[B122] Woolbright B. Jaeschke H. (2017). Role of the inflammasome in acetaminophen-induced liver injury and acute liver failure. J. Hepatol. 66, 836–848. 10.1016/j.jhep.2016.11.017 27913221 PMC5362341

[B123] Xu Q. Shi W. Lv P. Meng W. Mao G. Gong C. (2020). Critical role of caveolin-1 in aflatoxin B1-induced hepatotoxicity via the regulation of oxidation and autophagy. Cell Death Disease 11, 6 10.1038/s41419-019-2197-6 31919341 PMC6952418

[B124] Yang W. Lian J. Feng Y. Srinivas S. Guo Z. Zhong H. (2014). Genome-wide miRNA-profiling of aflatoxin B1-induced hepatic injury using deep sequencing. Toxicol. Lett. 226, 140–149. 10.1016/j.toxlet.2014.01.021 24472605

[B125] Yang X. Lv Y. Huang K. Luo Y. Xu W. (2016). Zinc inhibits aflatoxin B1-induced cytotoxicity and genotoxicity in human hepatocytes (HepG2 cells). Food Chem. Toxicol. 92, 17–25. 10.1016/j.fct.2016.03.012 27017951

[B126] Yener Z. Celik I. Ilhan F. Bal R. (2009). Effects of Urtica dioica L. seed on lipid peroxidation, antioxidants and liver pathology in aflatoxin-induced tissue injury in rats. Food Chem. Toxicol. 47, 418–424. 10.1016/j.fct.2008.11.031 19073231

[B127] Zhang L. Dou X. Zhang C. Logrieco A. Yang M. (2018). A review of current methods for analysis of mycotoxins in herbal medicines. Toxins 10, 65 10.3390/toxins10020065 PMC5848166 29393905

[B128] Zhang L. Zhan D. Chen Y. Wang W. He C. Lin Y. (2019). Aflatoxin B1 enhances pyroptosis of hepatocytes and activation of Kupffer cells to promote liver inflammatory injury via dephosphorylation of cyclooxygenase-2: an *in vitro*, *ex vivo* and *in vivo* study. Arch. Toxicol. 93, 3305–3320. 10.1007/s00204-019-02572-w 31612242

[B129] Zheng N. Zhang H. Li S. Wang J. Liu J. Ren H. (2018). Lactoferrin inhibits aflatoxin B1- and aflatoxin M1-induced cytotoxicity and DNA damage in Caco-2, HEK, Hep-G2, and SK-N-SH cells. Toxicon 150, 77–85. 10.1016/j.toxicon.2018.04.017 29753785

[B130] Zhu L. Gao J. Huang K. Luo Y. Zhang B. Xu W. (2015). miR-34a screened by miRNA profiling negatively regulates Wnt/β-catenin signaling pathway in Aflatoxin B1 induced hepatotoxicity. Sci. Rep. 5, 16732 10.1038/srep16732 26567713 PMC4645126

